# Characterization and *In Vitro* Skin Permeation of Meloxicam-Loaded Liposomes versus Transfersomes

**DOI:** 10.1155/2011/418316

**Published:** 2010-11-07

**Authors:** Sureewan Duangjit, Praneet Opanasopit, Theerasak Rojanarata, Tanasait Ngawhirunpat

**Affiliations:** Faculty of Pharmacy, Silpakorn University, Sanamchan Palace Campus, Nakhon Pathom 73000, Thailand

## Abstract

The goal of this study was to develop and evaluate the potential use of liposome and transfersome vesicles in the transdermal drug delivery of meloxicam (MX). MX-loaded vesicles were prepared and evaluated for particle size, zeta potential, entrapment efficiency (%EE), loading efficiency, stability, and *in vitro* skin permeation. The vesicles were spherical in structure, 90 to 140 nm in size, and negatively charged (−23 to −43 mV). The %EE of MX in the vesicles ranged from 40 to 70%. Transfersomes provided a significantly higher skin permeation of MX compared to liposomes. Fourier Transform Infrared Spectroscopy (FT-IR) and Differential Scanning Calorimetry (DSC) analysis indicated that the application of transfersomes significantly disrupted the stratum corneum lipid. Our research suggests that MX-loaded transfersomes can be potentially used as a transdermal drug delivery system.

## 1. Introduction

Transdermal drug delivery systems (TDDs) offer a number of potential advantages over conventional methods such as injectable and oral delivery [1]. However, the major limitation of TDDs is the permeability of the skin; it is permeable to small molecules and lipophilic drugs and highly impermeable to macromolecules and hydrophilic drugs. The main barrier and rate-limiting step for diffusion of drugs across the skin is provided by the outermost layer of the skin, the stratum corneum (SC) [2]. Several strategies have been developed to overcome the skin's resistance, including the use of prodrugs, ion pairs, liposomes, microneedles, ultrasound, and iontophoresis [3–6].

Various types of liposomes (LPs) exist, such as traditional liposomes, niosomes, ethosomes, and transfersomes [3, 7–12]. Various LPs have been extensively investigated for improving skin permeation enhancement. Liposomes are promising carriers for enhancing skin permeation because they have high membrane fluidity. Previous reports indicate that liposomes can deliver a large quantity of hydrophilic drugs (e.g., sodium fluorescein [13], carboxyfluorescein [14]), lipophilic drugs (e.g., retinoic acid [11], tretinoin [12]), proteins, and macromolecules through the skin. Many factors influence the percutaneous penetration behavior of LPs, including particle size, surface charge, lipid composition, bilayer elasticity, lamellarity, and type of LP [7, 12]. 

Cevc's group introduced Transfersomes, which are the first generation of elastic vesicles. Transfersomes are prepared from phospholipids and edge activators. An edge activator is often a single-chain surfactant with a high radius of curvature that destabilizes the lipid bilayers of the vesicles and increases the deformability of the bilayers. Sodium cholate, sodium deoxycholate, Span 60, Span 65, Span 80, Tween 20, Tween 60, Tween 80, and dipotassium glycyrrhizinate were employed as edge activators. Compared with subcutaneous administration, transfersomes improved *in vitro* skin permeation of various drugs, penetrated intact skin *in vivo*, and efficiently transferred therapeutic amounts of drugs [9, 15, 16]. However, the mechanism by which LPs and their analogs deliver drugs through the skin is not fully understood [14]. 

**Figure 1:**
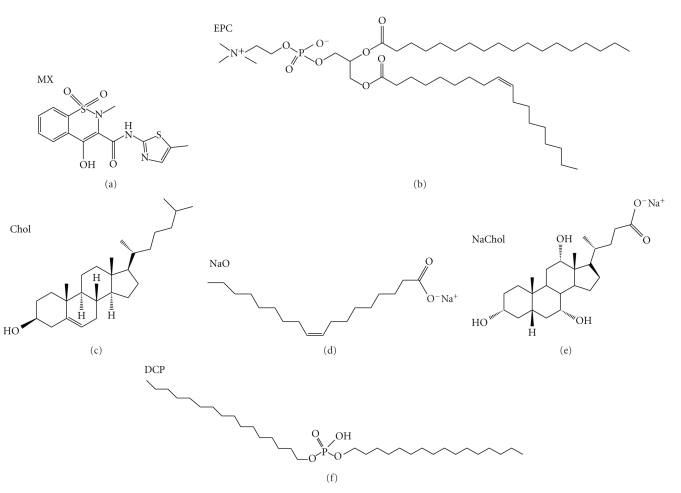
The chemical structure of meloxicam and the lipid compositions of the liposomes.

Meloxicam (Figure 1) has low aqueous solubility, and it is a highly potent, nonsteroidal anti-inflammatory drug (NSAID) that is used for treatment of rheumatoid arthritis and osteoarthritis [6, 17–19]. MX shows similar efficacy for reducing pain and inflammatory symptoms, but it has lower toxicity than other NSAIDs. Although MX is relatively potent and safe, its limitations include low solubility, low incorporation in formulations, and low skin permeation [6, 18–25]. In this study, vesicles were used as a novel MX transdermal drug delivery system. The system was developed and evaluated for its physicochemical characteristics, such as particle size, surface charge, entrapment efficiency, loading efficiency, stability, and *in vitro* skin permeation. The type of vesicles (liposomes and transfersomes), the composition of lipid in the liposomes (cholesterol), and transfersomes (cholesterol and surfactants) were evaluated. Three surfactants that differ in length of carbon chains were used for the preparation of transfersomes: sodium oleate (NaO, C_18_), sodium cholate (NaChol, C_24_), and dicetylphosphate (DCP, C_32_). Characterization of skin permeation was performed using FTIR and DSC. Figure 1 shows the chemical structure of meloxicam and the lipid compositions of the liposomes.

## 2. Materials and Methods

### 2.1. Materials

Phosphatidylcholine (PC) from eggs was purchased from GmbH. Cholesterol (Chol) was purchased from Carlo Erba Reagenti. Sodium cholate (NaChol) was purchased from Acros Organics. Sodium oleate (NaO) and dicetylphosphate (DCP) were purchased from Sigma-Aldrich. Meloxicam (MX) was supplied from Fluka.

### 2.2. Preparation of Meloxicam-Loaded Liposomes, Transfersomes, and Suspensions

Liposomes containing a controlled amount of PC and various amounts of MX were formulated. The MX concentration was varied from 2.5 to 70.0 wt. % of the PC. The sonication method was used to prepare different formulations; they were composed of bilayer-forming PC and either Chol, NaO, NaChol, or DCP in a molar ratio of 10 : 2. The PC, Chol, NaO, NaChol, DCP, and MX were each briefly dissolved in chloroform:methanol (2 : 1 v/v). In preparing MX-loaded liposomes and transfersomes, the materials were deposited in a test tube, and the solvent was evaporated with nitrogen gas. The lipid film was placed in a desiccator connected to a vacuum pump for a minimum of 6 h to remove the remaining organic solvent. The dried lipid film was hydrated with Tris buffer. Following hydration, the dispersion was sonicated in a bath for 30 min and then probe-sonicated for 2 cycles of 30 min. The lipid compositions of the different formulations utilized in this study are listed in Table 1.

For the preparation of MX suspensions, the saturated solubility of MX in water was determined to ensure excess drug in MX suspension. The solubility of MX was determined by adding excess amount of MX to 5 mL of water in a glass vial and stirring by a magnetic stirrer for 24 h. The sample was filtered through 0.45 *μ*m membrane filter in order to remove undissolved drugs in the saturated solution. The concentration of MX was analyzed by HPLC. The MX suspension was prepared by adding MX to distilled water at a concentration 2 times higher than the solubility of MX and stirring for 24 h to ensure constant thermodynamic activity throughout the course of the permeation experiment. The particle size of MX suspension was determined, and the MX suspension was used in the skin permeation experiment.

### 2.3. Characterization of Liposomes and Transfersomes

#### 2.3.1. Particle Size and Surface Charge

The droplet size and zeta potential of the liposomes and transfersomes were determined by a Laser Scattering Particle Size Distribution Analyzer and Zeta Potential Analyzer at room temperature. One mL of the liposome and transfersome suspensions were diluted with 14 mL and 2 mL deionized water, respectively.

#### 2.3.2. Transmission Electron Microscopy

Transmission Electron Microscopy (TEM) was used to visualize the liposomal and transfersomal vesicles. The vesicles were dried on a copper grid and adsorbed with filter paper. After drying, the sample was viewed under the microscope at 10–100 k magnification at an accelerating voltage of 100 kV.

#### 2.3.3. Entrapment Efficiency (%EE) and Loading Efficiency

The concentration of MX in the formulation was determined by HPLC analysis after disruption of the vesicles (liposomes and transfersomes) with Triton X-100 (0.1% w/v) at a 1 : 1 volume ratio and appropriate dilution with PBS (pH 7.4). The vesicle/Triton X-100 solution was centrifuged at 10,000 rpm at 4°C for 10 min. The supernatant was filtered with a 0.45 *μ*m nylon syringe filter. The entrapment efficiencies and the loading efficiencies of the MX-loaded formulation were calculated by (1) and (2), respectively.

(1) $$\text{\%}\;\text{entrapment}\;\text{efficiency}=\;\left(\frac{C_{L}}{C_{i}}\right)\times 100,$$

where *C*_*L*_ is the concentration of MX loaded in the formulation as described in the above methods, and *C*_*i*_ is the initial concentration of MX added into the formulation

(2) $$\text{loading}\;\text{efficiency}=\frac{D_{t}}{L_{t}},$$

where *D*_*t*_ is the total amount of MX in the formulation and *L*_*t*_ is the total amount of PC added into the formulation.

#### 2.3.4. Stability Evaluation of Liposomes and Transfersomes

Liposomes and transfersomes were stored at 4 ± 1°C and 22 ± 1°C (room temperature, RT) for 30 days. Both the physical and the chemical stability of MX were evaluated. The physical stability was assessed by visual observation for sedimentation and particle size determination. The chemical stability was determined by measuring the MX content by HPLC on days 0, 1, 7, 14, and 30.

### 2.4. In Vitro Skin Permeation Study

Shed snake skin from the Siamese cobra (*Naja kaouthia*) was used as a model membrane for the skin permeation study because of its similarity to human skin in lipid content and permeability. The skin samples were mounted between the two half-cells of a side-by-side diffusion chamber with a 37°C water jacket to control the temperature. The dorsal surface of the skin was placed in contact with the donor chamber, which was filled with the liposome formulation. The receptor chamber was filled with 0.1 M PBS (pH 7.4) and stirred with a star-head Teflon magnetic bar driven by a synchronous motor. At time intervals of 0.5, 1, 2, 4, 8 and, 24 h, a 1 mL aliquot of receptor was withdrawn, and the same volume of fresh medium was added back into the chamber. The concentration of MX in the samples was analyzed by HPLC. The concentration of permeants in the samples was analyzed by HPLC, and the cumulative amount was plotted against time. The steady-state flux was determined as the slope of linear portion of the plot. Lag time was also obtained by extrapolating the linear portion of the penetration profile to the abscissa.

### 2.5. HPLC Analysis

The MX concentration was analyzed by HPLC [28] using an Eclipse XDB-C18 column. The mobile phase was a mixture of potassium dihydrogen phosphate pH 4.4, methanol, and acetonitrile at a ratio of 45 : 45 : 10 (v/v/v). A 20 *μ*L injection volume was used with a flow rate of 1.0 mL/min, and UV detection was viewed at 364 nm. The quantitative determination of MX in the tested sample was obtained from the calibration curve, which gave good linearity at the range of 0.1–50 *μ*g/mL.

### 2.6. Characterization of Snake Skin after Skin Permeation

#### 2.6.1. FT-IR Analysis of Shed Snake Skin

Following the skin permeation study, the skin was washed with water and blotted dry by keeping in the desiccator for 24 h. The spectrum of the snake skin was recorded in the range of 4000–500 cm^−1^ using an FT-IR spectrophotometer. The FT-IR spectrum of the untreated skin was also recorded and used as a control.

#### 2.6.2. Differential Scanning Calorimetry (DSC) Analysis of Shed Snake Skin

Thermal analysis of the skin after the permeation study prepared with the same method as FTIR was performed with a Sapphire DSC. The skin sample (2 mg) was weighed into an aluminum crimp pan. The samples were heated from −30 to 320°C at a heating rate of 10°C/min. All DSC measurements were collected under a nitrogen atmosphere with a flow rate of 100 mL/min.

### 2.7. Data Analysis

Data are expressed as the means ± standard deviation (SD) of the mean, and statistical analysis was carried out employing the one-way analysis of variance (ANOVA) followed by an LSD *post hoc *test. A value of *P* < .05 was considered statistically significant.

## 3. Results and Discussion

### 3.1. Physicochemical Characteristics of Liposomes and Transfersomes

The particle size range for all formulations, except the MX suspensions, was less than 200 nm (89 to 137 nm) with a narrow size distribution. The particle size range of the MX suspensions was significantly larger than that of the liposomes (Table 2). The vesicles containing cholesterol had a slightly lower particle size than without cholesterol. These results might be attributed to cholesterol causing the bilayer to be more compact [10, 26, 29–31]. The particle size of the transfersomes with different types of surfactant did not show a significant difference. These results indicated that the particle size of the vesicles was not affected by lipid composition (cholesterol) and surfactant. 

The zeta potential of all vesicle formulations were negative (−23 to −43 mV) due to the net charge of the lipid composition in the formulations. PC is a zwitterionic compound with an isoelectric point (pI) between 6 and 7 [32]. Under experimental conditions (pH 7.4), where the pH was higher than its pI, PC carried a net negative charge. The surfactants used were anionic surfactants, and the anion form of MX was also the predominant form at pH 7.4 [25]. Therefore, a negative charge in all formulations was observed. Because the negatively charged liposome formulations strongly improved skin permeation of drugs in transdermal delivery [12], these formulations were chosen to be tested for MX permeation in our study. 

**Figure 2:**
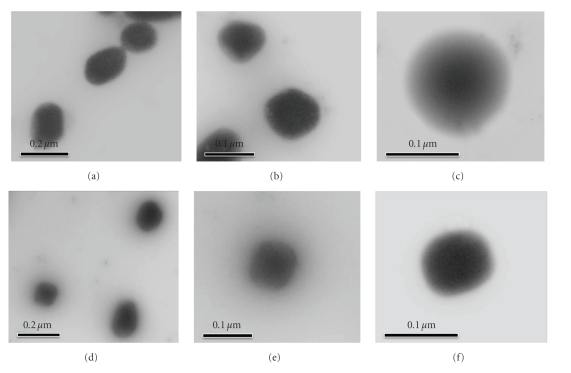
Transmission electron microscopy of MX loaded in vesicles. (a) visualization of MX loaded in liposomes (PC) (10,000x), (b) visualization of MX loaded in liposomes (PC) (30,000x), (c) visualization of MX loaded in liposomes (PC) (50,000x), (d) Visualization of MX loaded in transfersomes (PC/NaChol) (10,000x), (e) visualization of MX loaded in transfersomes (PC/NaChol) (30,000x), and (f) visualization of MX loaded in transfersomes (PC/NaChol) (50,000x).

The morphology of the two-dimensional vesicles was further evaluated by TEM, justifying the vesicular characteristics. MX loaded in liposomes prepared from PC and PC/NaChol was spherical in shape (Figures 2(a), 2(b), and 2(c)) and spherical with unilamellar vesicles (Figures 2(d), 2(e), and 2(f)), respectively.

### 3.2. Entrapment Efficiency and Loading Efficiency

**Figure 3:**
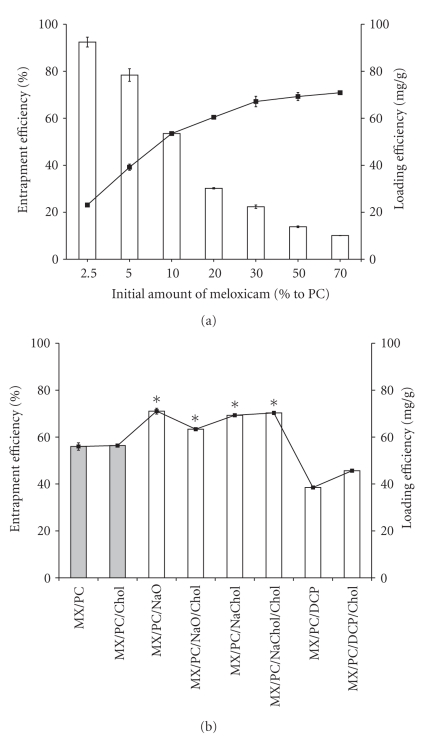
(a) The effect of initial amount of meloxicam (2.5, 5, 10, 20, 30, 50, and 70%) added in liposomes on percentage entrapment efficiency (white bar) and loading efficiency (fill square) of meloxicam loaded in liposomes composed of PC. Each value represents the mean ± SD (*n* = 3) (b) The percentage entrapment efficiency (white bar) and loading efficiency (fill square) of meloxicam loaded in different formulations: (shaded square) liposomes and (white square) transfersomes. Each value represents the mean ± SD (*n* = 6).

The entrapment efficiencies and loading efficiencies of the MX-loaded formulations are presented in Figure 3(a). The 2.5% MX-LP formulation had the highest entrapment efficiency but the lowest loading efficiency, while the 70% MX-LP formulation showed the highest loading efficiency but the lowest entrapment efficiency. Therefore, there should be an optimum ratio between PC and MX for developing MX-loaded vesicles as carriers for transdermal drug delivery. The optimum ratio, which offered high entrapment efficiency and high loading efficiency, was 10% MX-LP. This ratio was used to prepare the vesicles.

The entrapment efficiency and loading efficiency of transfersome formulations were significantly higher than the liposome formulations (Figure 3(b)). The entrapment efficiency of MX in the vesicles ranged from 38% to 71%. The entrapment of MX in liposomes was lower than transfersomes except in formulations with DCP. This result might be attributed to interactions between the surfactants (NaO and NaChol) and MX when the complex was inserted into the transfersomes bilayer. Fang et al. reported that adding surfactant (sodium stearate) to phosphatidylethanolamine vesicles significantly increased the entrapment efficiency of 5-aminolevulinic acid [26]. The results indicated that the type of carrier systems and lipid composition affected the entrapment efficiency and loading efficiency of MX in the vesicle formulations.

The entrapment efficiency of the vesicles with and without cholesterol did not show a significant difference. However, the entrapment efficiencies of the transfersome formulations changed depending on the type of surfactant used and ranked PC/NaO(C_18_)>PC/NaChol (C_24_)>PC/DCP(C_32_). The lower the carbon chain length of the surfactants in the formulation, the higher the entrapment efficiency. The increase in the carbon chain length of the surfactant increased the lipophilicity and the solubility of lipophilic drug in the bilayer [10, 27]. This characteristic may explain the increase in entrapment efficiency of MX in the bilayer of the vesicles. Surfactant may also compete with MX when arranging in the bilayer and therefore exclude the drug as it assembles into the bilayer of the vesicles. The data indicated that the entrapment efficiency and loading efficiency are independent of cholesterol but dependent on the surfactant in the formulations.

### 3.3. Stability Evaluation of Liposomes and Transfersomes

**Figure 4:**
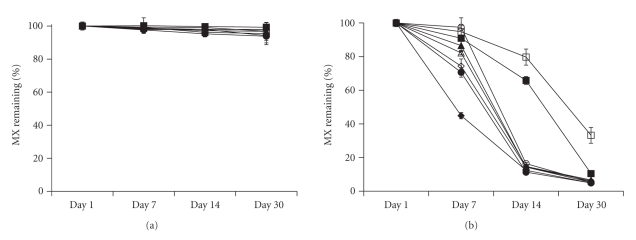
The percentage of meloxicam remaining in vesicles composed of different compositions: (solid diamond) PC, (white diamond) PC/Chol, (solid triangle) PC/NaO, (white triangle) PC/NaO/Chol, (solid circle) PC/NaChol, (white circle) PC/NaChol/Chol, (solid square) PC/DCP, and (white square) PC/DCP/Chol following storage at (a) 4°C and (b) RT for 30 days. Each value represents the mean ± SD (*n* = 3).

Liposomes and transfersomes were stored at 4°C or RT for 30 days. The physical (particle size determination) and chemical (percent MX remaining in the formulation) stability of the vesicles are presented in Table 3 and Figure 4, respectively. No sedimentation was found in any vesicle formulation after fresh preparation. After storage at 4°C for 30 days, there was no sedimentation, but the average size of the vesicles in all formulations slightly increased. Nevertheless, the average size remained under 200 nm (Table 3). After storage at RT for 7 days, no sedimentation was present in any formulation (data not shown). When evaluating the chemical stability of the vesicles, the percentage of MX remaining at 4°C for 30 days was in the range of 93% to 99% (Figure 4(a)), but it was 4% to 33% for the samples at RT (Figure 4(b)). The degradation rate of the MX-loaded vesicles stored at 4°C was not significantly different than those that were freshly prepared. This reveals that the degradation of MX is independent of lipid composition but dependent on the storage temperature and age.

### 3.4. In Vitro Skin Permeation Study

**Figure 5:**
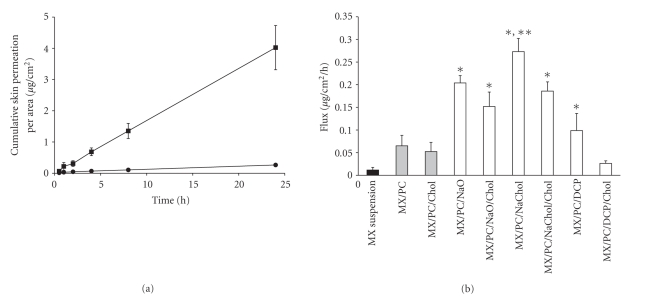
(a) The skin permeation profile of meloxicam from (solid circle) MX suspensions (control) and (solid square) MX/PC/NaChol. (b) The fluxes of meloxicam through shed snake skin from different formulations: (solid square) control, (shaded square) liposomes, and (white square) transfersomes. Different values ∗  were statistically significant (*P* < .05) compared with MX suspensions (control). Different values ∗∗ were statistically significant (*P* < .05) compared with liposomes. Each value represents the mean ± SD (*n* = 3–6).

Figure 5(a) illustrates the permeation profiles of MX suspensions (control) and MX-loaded transfersomes with NaChol. The cumulative amount of drug increased linearly with time after a short lag time (0.5–0.8 h). This linear accumulation was also observed for other formulations (data not shown).  Figure 5(b) shows the flux (F) of MX through the snake skin calculated from the permeation profiles. The F of MX permeated through the skin in all vesicle formulations was significantly higher than the MX suspensions. The vesicle systems were able to promote skin permeation of an active drug by a variety of mechanisms: (a) the free drug mechanism, (b) the penetration-enhancing process of the liposome components, (c) vesicle adsorption to and/or fusion with the SC, and (d) intact vesicle penetration into and through the intact skin and the localization at the site of action [33–35]. Moreover, the similar predominance to the lipid bilayer of biological membranes [36] and the nanometer size range of the vesicles may be also influenced [7, 26, 30]. These results indicated that the vesicle system can overcome the barrier function of the stratum corneum by various mechanisms and their physicochemical properties.

The F of MX permeated through the skin in transfersomes was significantly higher than in liposomes. Transfersomes have shown to be successful in the delivery of drugs into the skin, including diclofenac, triamcinolone acetonide, hydrocortisone, and estradiol. Because transfersomes are composed of PC and surfactants, they can squeeze through the pores in the SC, which are smaller than one-tenth their diameter [3]. They can also adsorb onto or fuse with the SC, and the intact vesicle can penetrate into and through the intact skin. 

The F of MX in the vesicles composed of cholesterol was slightly lower than vesicles without cholesterol. An increase in cholesterol could lead to increased stability and rigidity and decrease the permeability of the lipid bilayer, which may cause lower release of MX and lower permeation of MX through the skin [31]. The F of MX permeated from transfersomes with different compositions of surfactants are ranked as follows: NaO (C_18_)~NaChol (C_24_)>DCP (C_32_). The lower the carbon chain length of the surfactant in the formulation, the higher the skin permeation of MX. The particle size and %EE of the vesicles composed of NaO and NaChol were smaller and higher than vesicles containing DCP, respectively. These results indicated that the barrier function of stratum corneum can be overcome by several factors, including physicochemical properties of vesicle systems (size, charge, and %EE), lipid composition (cholesterol, surfactant), and type of vesicle system (liposomes, transfersomes).

The research results indicated that the skin permeability of MX-loaded transfersomes and liposomes were greater than that of MX suspensions and that both PC and surfactant were key factors. Surfactants are enhancers that solubilize the lipophilic compound; they also have the potential to solubilize the lipid within the SC. Surfactants swell the SC, interact with the intercellular keratin, and fluidize the SC lipid to create channels that allow increased drug delivery.

### 3.5. Characterization of the Skin

**Figure 6:**
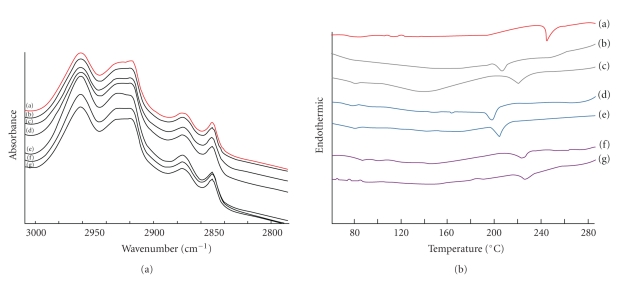
(a) FT-IR spectra profile of shed snake skin after 24 h transfersomes skin permeation. (a) Untreated skin, (b) PC/NaO, (c) PC/NaO/Chol, (d) PC/NaChol, (e) PC/NaChol/Chol, (f) PC/DCP, and (g) PC/DCP/Chol and (b) DSC thermogram of shed snake skin after 24 h MX suspensions (control) and transfersomes skin permeation. (a) MX suspensions, (b) PC/NaO, (c) PC/NaO/Chol, (d) PC/NaChol, (e) PC/NaChol/Chol, (f) PC/DCP, and (g) PC/DCP/Chol.

The FT-IR spectrum of the snake skin as a model for the SC provided a measure of fluidity of the SC lipid. The comparison of the spectral profile of the untreated skin and treated skin with transfersomes, with and without cholesterol, resulted in shifts to higher frequencies. There was an absorbance broadening for both the C–H (CH_2_) asymmetric stretching peak near 2920 cm^−1^ and the C–H (CH_2_) symmetric stretching peak near 2850 cm^−1^ (Figure 6(a)) [37]. The data indicated that flexibility of the SC lipid upon application of transfersomes occurred. Thus, it can be hypothesized that transfersomes permeated through the skin by disruption of the SC lipid structure.

The disruption of the SC lipid by the application of transfersomes was further evaluated by DSC (Figure 6(b)). The SC lipid of the snake skin exist as a solid gel at temperature of 244°C. In the DSC study, when the skin was treated with transfersomes, which exists as liquid state vesicles, their thermal properties shifted (melting point; Tm) as follows: PC/NaChol, 198°C; PC/NaO, 207°C; PC/DCP, 218°C; PC/NaChol/Chol, 207°C; PC/NaO/Chol, 222°; PC/DCP/Chol, 221°C. The data indicated that the *T*_*m*_ of skin treated with transfersomes was significantly lower than that of the untreated skin. The change into lower transition temperature suggests an increase in the gross fluidity of the SC lipids. This is consistent with the general view that the mechanism of action of the surfactant is attributed to the alteration of the lipid organization and an increase in lipid lamellae disorder in the SC. Moreover, the *T*_*m*_ of the skin treated with transfersomes with cholesterol was significantly higher than those without cholesterol. If cholesterol could be complexed with phospholipids in the skin, it could add more structure to the bilayer. These results were in accordance with skin permeation data showing that transfersomes increased the skin permeation of MX, and the addition of cholesterol in the transfersomes also led to a decrease in skin permeation of MX when compared with transfersomes without cholesterol. Transfersomes may be used as alternative carriers for transdermal drug delivery potential because they interact with solid gel phase SC lipids and thus leading to disruption and fluidization of the SC lipid.

## 4. Conclusion

In the present study, MX-loaded transfersomes were successfully prepared by a sonication method. The use of surfactants containing medium length carbon chains, including NaO (C_18_) and NaChol (C_24_), in the transfersomes resulted in a high entrapment efficiency. Transfersomes provide greater MX skin permeation than liposome and MX suspensions. The mechanism of this increase in MX permeation may be through transfersomes' disruption of the SC lipid. The data indicate that the barrier function of SC was affected by several factors, including physicochemical properties of vesicle systems (size, charge, %EE), lipid composition (cholesterol, surfactant), and type of vesicle system (liposomes, transfersomes). Our research suggests that utilizing MX-loaded transfersomes as a transdermal therapeutic agent shows potential.

## Figures and Tables

**Table 1:** The lipid compositions of the different formulations used in study.

Name (molar ratio)	Composition (%W/V)						
	MX	PC	Chol	NaO	NaChol	DCP	PBS ph 7.4
MX/PC (2 : 10)	0.07	0.77	—	—	—	—	100 mL
MX/PC/Chol (2 : 10 : 2)	0.07	0.77	0.07	—	—	—	100 mL
MX/PC/NaO (2 : 10 : 2)	0.07	0.77	—	0.06	—	—	100 mL
MX/PC/NaO/Chol (2 : 10 : 2 : 2)	0.07	0.77	0.07	0.06	—	—	100 mL
MX/PC/NaChol (2 : 10 : 2)	0.07	0.77	—	—	0.08	—	100 mL
MX/PC/NaChol/Chol (2 : 10 : 2 : 2)	0.07	0.77	0.07	—	0.08	—	100 mL
MX/PC/DCP (2 : 10 : 2)	0.07	0.77	—	—	—	0.11	100 mL
MX/PC/DCP/Chol (2 : 10 : 2 : 2)	0.07	0.77	0.07	—	—	0.11	100 mL

**Table 2:** Particle size and zeta potential in various formulations.

Name	Particle size (nm)	Zeta potential (mV)
MX suspensions	2411 ± 84.2	−19.3 ± 0.7
MX/PC	107.0 ± 5.0	−35.0 ± 0.5
MX/PC/Chol	100.3 ± 0.6	−23.5 ± 0.2
MX/PC/NaO	107.4 ± 0.5	−43.4 ± 0.1
MX/PC/NaO/Chol	100.5 ± 0.6	−23.1 ± 0.0
MX/PC/NaChol	93.0 ± 1.0	−32.7 ± 0.7
MX/PC/NaChol/Chol	88.6 ± 0.7	−28.9 ± 0.5
MX/PC/DCP	137.2 ± 6.1	−35.2 ± 0.6
MX/PC/DCP/Chol	126.5 ± 1.6	−29.3 ± 0.5

Each value represents the mean ± SD (*n* = 3).

**Table 3:** Particle size of formulations composed of different formulations following storage at 4°C for 30 days.

Name	Practicle size (nm)				
	Day 0	Day 1	Day 7	Day 14	Day 30
MX/PC	107.0 ± 5.0	113.4 ± 4.3	114.0 ± 1.1	114.5 ± 3.7	126.9 ± 16.0
MX/PC/Chol	100.3 ± 0.6	130.3 ± 15.5	159.0 ± 1.2	163.1 ± 2.5	182.6 ± 4.5
MX/PC/NaO	107.4 ± 0.5	93.8 ± 2.3	91.7 ± 0.9	93.8 ± 6.9	97.4 ± 2.0
MX/PC/Nao/Chol	100.5 ± 0.6	99.9 ± 1.1	96.1 ± 1.2	100.5 ± 5.5	110.6 ± 25.7
MX/PC/NaChol	93.0 ± 1.0	93.0 ± 1.0	93.6 ± 2.0	94.5 ± 1.6	92.1 ± 2.1
MX/PC/NaChol/Chol	88.6 ± 0.7	74.0 ± 2.5	87.4 ± 7.8	85.4 ± 4.3	85.1 ± 2.0
MX/PC/DCP	137.2 ± 6.1	144.5 ± 6.8	152.4 ± 1.2	162.3 ± 2.9	162.0 ± 4.9
MX/PC/DCP/Chol	126.5 ± 1.6	131.6 ± 3.9	139.5 ± 2.8	166.3 ± 12.9	184.9 ± 3.0

Each value represents the mean ± SD (*n* = 3).
